# Economic analysis of youth participation in agripreneurship in Benin

**DOI:** 10.1016/j.heliyon.2022.e08738

**Published:** 2022-01-10

**Authors:** Rexford Akrong, Bekele Hundie Kotu

**Affiliations:** a University of Cape Coast, Department of Integrated Development Studies, Cape Coast, Ghana; b International Institute of Tropical Agriculture (IITA), Tamale, Ghana

**Keywords:** Agribusiness, Agripreneurship, Cash crop, Entrepreneurship, Youth

## Abstract

The study assessed the factors affecting youth participation in rural entrepreneurship in Benin using data from the School-to-Work Transition Survey (SWTS) and applying the binary logit and the multinomial logit models. The results showed youth who have a larger number of children are more likely to choose agricultural businesses (agripreneurship) while those who have formal education, who have received training on entrepreneurship, who have registered business, and those who have located in urban areas are more likely to engage in non-agricultural businesses. Within agripreneurship, youth who belong to a larger household are more likely to engage in farming while those who are educated, who have access to credit, and who are located in urban areas are more likely to be engaged in non-farming agri-businesses. The study also revealed that cash crop production among Beninese youth was positively influenced by access to credit. The findings suggest that it would be necessary to promote development programmes that are geared towards enhancing the capacities of the youth with regards to concepts and skills of entrepreneurship in agriculture and measures to overcome challenges associated with different agribusiness activities.

## 1 Introduction

Achieving the eighth Sustainable Development Goal (SDG 8) of inclusive and sustainable economic growth, employment and decent work for all requires devising strategies that are critical to providing new employment opportunities for all persons. This includes making seemingly unattractive but lucrative sectors of economies of developing countries attractive and more lucrative to all persons. Agriculture presents several employment opportunities to the African youth (people in the age bracket of 15–35) (African Union, 2006) whose population is estimated to grow by 40 percent by 2030 (African Development Bank, 2016). Agriculture can also be an avenue for income generation, poverty reduction and improvement in food and nutrition security for this group of the population (Kidodo et al., 2016). Thus, agriculture is a pathway to youth empowerment. Therefore, making the agricultural sector attractive and lucrative implies achieving the first, second and eighth SDGs of no poverty, zero hunger, and decent work and economic growth.

The importance of agriculture to the economy of Benin cannot be overemphasized. The sector is a source of livelihood and employment to about 70–80 percent of the country's population (Adjimoti, 2018), provides foreign exchange earnings and food and nutrition security (USDA, 2014). These benefits call for investment in the agricultural sector as the key driver of the country's economic growth (Karimou, 2018).

Despite the significant contribution of agriculture to the economy of Benin, the sector is fraught with some serious constraints such as inadequate input supply, high dependence on rainfall, land tenure (lack of land title), distrust among farmers, pests and diseases, and inadequate access to finance. These challenges coupled with the negative perceptions of youth, who form about 60 percent of the population, about agriculture being less lucrative, labor and capital intensive, and an activity with low self-esteem make agriculture unattractive to the youth, hence their low participation in agriculture (IFAD, 2019; Mangal, 2009; Yami et al., 2019). Meanwhile, youth engagement in agriculture has been found to increase agricultural productivity given that this group is in the physical and mental primes of their lives, are flexible and dynamic, and are relatively more educated than the elderly population (Mangal, 2009; Naamwintome and Bagson, 2013). Furthermore, youth participation in agriculture is important in replacing the elderly population in agriculture, decrease imports of staple food, reduce the poor image of agriculture, reduce rural-urban migration and reduce youth unemployment and its associated social problems (Naamwintome and Bagson, 2013; Twumasi et al., 2019). Regarding poverty reduction, Osabohien et al. (2021) found that youth participation in agriculture can reduce poverty by 17 percent. Thus, there is a need for agricultural transformation programmes to boost the engagement of this group in agriculture (Osabohien et al., 2020).

However, over the past decades, West Africa has seen a phase of rising cases of youth unemployment which is more pronounced in urban areas. Studies have identified that key drivers of the problem of youth unemployment particularly in urban areas include job-skills mismatch (Morsy and Mukasa, 2019) and the influx of rural youth in urban centers through rural-urban migration, which has increased the supply of labour. Meanwhile, there are limited formal employment opportunities to absorb this increasing labour force in Benin. To curb the incidence of unemployment in Benin and West Africa at large, government and development partners alike have emphasised the need for entrepreneurship. Agripreneurship (i.e., entrepreneurship in agriculture) has been identified as a major pathway to increase employment among rural youth, thereby decreasing the incidence of rural-urban migration of youth and its spillover effects. This is due to the employment potential of the agricultural sector given that this sector employs nearly 80 percent of the Beninese population (Adjimoti, 2018). To this end, several studies in different countries have examined the willingness of youth to participate in agricultural training programs, pursue agriculture in school or pursue agribusiness as well as the drivers of youth participation in agriculture and agribusiness (Adeyanju et al., 2021; Haruna et al., 2019; Magagula and Tsvakirai, 2020; Ng'atigwa et al., 2020; Twumasi et al., 2019). However, there is limited information on drivers of youth choices of different agripreneurship activities as well as their crop production decisions in Benin. To this end, this study extends the literature by assessing what drives youth to participate in the different nodes of agribusiness as well as the production of different categories of crops in Benin. Such information is necessary to devise policies regarding institutions and infrastructure that are critical to empowering these vulnerable members of the population through agriculture.

The rest of the paper is structured in the following order: Section 2 presents the literature review. Section 3 presents the data sources and methods of data analysis. Section 4 presents the results and discussions and Section 5 presents the conclusion and policy implication of the study.

## 2 Literature review

Youth participation in agriculture or agripreneurship has received attention on the academic front. Researchers have modelled the single-step decision involved in youth agripreneurship decisions using the binary logit or probit models. Nwibo et al. (2016) used the binary logit model to assess the determinants of agripreneurship among rural households of Ishielu Local Government Area of Ebonyi State. The study found a negative relationship between age and the decision to become an agripreneur and a positive relationship between household size, educational status, annual income and agripreneurship experience positively influenced the decision to be an agripreneur. This study informed the current study on the variables to include in the econometric model. However, this study did not consider the different agripreneurship activities. The current study considered the different agripreneurship activities in which rural youth are involved.

Ogunmodede et al. (2020) employed the binary logit model to estimate the factors that influence the choice of Nigerian youth to create employment through agriculture. The study found that the factors that positively influence youth to become agripreneurs include age and agribusiness years of experience whereas education and being employed reduced the propensity of being an agripreneur. This study informed the current study on the need to model the choice of Beninese youth to be entrepreneurs in agriculture. However, this study did not delineate to examine the choice of agripreneurship activity.

Magagula and Tsvakirai (2020) examined the factors that influence the intention of youth to participate in agripreneurship in South Africa. The findings of the study revealed parental financial support, pursuing agricultural studies in school and perceived economic benefits accrued to agriculture positively influence a youth's intention to pursue agripreneurship whereas marital status negatively influenced a youth's intention to pursue agriculture. Although this study does not consider what factors influence the decision to participate in agriculture, it provides information on the factors that influence a youth's willingness to participate in agripreneurship. This study differs from the present study in geographical terms. South Africa has a relatively more sophisticated and advanced agricultural sector compared with Benin. Thus, the information from the present study is necessary to provide evidence on agripreneurship decisions in the context of a developing agricultural sector in the context of a lower-middle-income country.

Ng'atigwa et al. (2020) used the ordered logit model to analyse youth participation in horticulture agribusiness in Tanzania. The study revealed that education, management innovation, access to credit, good perception of horticulture for agribusiness and improved packaging materials positively influenced youth engagement in horticulture agribusiness. However, gender and land size negatively influenced youth participation in horticulture agribusiness in Tanzania. This study did not consider the different aspects of horticulture agribusiness (farming and trading). However, the current study considers the different activities involved in agribusiness given that engagement in these activities yields different levels of utility (profit).

Applying the double hurdle model, Twumasi et al. (2019) assessed the factors that influence participation and the intensity of tertiary youth participation in agriculture in Ghana. The results revealed that the perceived price of farm inputs, access to credit, access to land, education, and agricultural studies influenced youth to participate in agriculture. On the other hand, whereas the perceived price of farm input and being a male reduced the intensity of participation, access to credit, access to land, youth course of study, and perceived benefits from agriculture influenced the intensity of participation. This study informed the current study on the stepwise nature of youth agripreneurship decisions. The present study advanced the concept of youth agripreneurship decisions to include more steps compared with Twumasi et al. (2019).

On the impact of agricultural programmes on youth participation in agribusiness, Adeyanju et al. (2021) used the endogenous switching probit model and found that youth participation in agricultural programmes was positively influenced by age, education, migration status, perception about training and mental health whereas being formally employed negatively influenced participation in such programmes. On the other hand, the results show that participation in agribusiness was positively influenced by gender, wealth and access to credit whereas education and mental health reduced the propensity to participate in agribusiness. This study informed the hypotheses of the current study by showing how the aforementioned variables influence youth participation in agribusiness. However, this study does not extend to show how different agribusiness activities are influenced by these variables.

This review of literature has revealed theoretical underpinnings of the concept of agripreneurship as well as models used to analyse agripreneurship decisions. Moreover, the review of recent empirical works on youth agripreneurship decisions has shown that several factors influence youth agriprepreneurship decisions in different countries with different socioeconomic, political and geographical conditions. However, none of these studies have considered the factors that influence Beninese youth's choice of different agriprenuership activities. To this end, a study of this nature was necessary to examine youth agripreneurship decisions in the context of different agripreneurship activities.

## 3 Methodology

### 3.1 Data

The data for this study were gathered from the School-to-Work Transition Survey (SWTS) (ILO, 2015). The SWTS generates relevant labour market information on youth aged between 15 to 29 including longitudinal information on transitions within the labour market. Although this age group is inconsistent with AU's definition of youth, the consideration of this age group in this study is largely due to data limitations. In Benin, the SWTS was implemented from December 2014 to January 2015 by the Institut National de la Statistique et de l’Analyse Economique (INSAE) in collaboration with the International Labour Organization (ILO) and the MasterCard Foundation, under the “Work4Youth” project. The data is nationally representative of individuals 15–29 years old. The sampling frame for the survey was a list of all households in Benin obtained from the Institut National de la Statistique et de l’Analyse Economique (INSAE). From the sampling frame, all youth in the randomly selected households were eligible for the survey. Eligible respondents (youth) from households in total 4,306 of Benin participated in the survey. The questionnaire used in the survey contains six sections for collecting quality information on youth. This information includes household demographic characteristics, formal education/training, activity history and aspirations, youth workers, non-working youth and youth not in the labour force (i.e., youth who were still in school at the time of the study). Generally, these participants include both employed and unemployed youth; however, this study was interested in entrepreneurs. Thus, out of the 4,306 youth who participated in the survey, this study identified respondents who were self-employed at the time of the survey. The result was a total sample of 765 youth entrepreneurs who were engaged in all sectors of the economy. These sampled entrepreneurs included agripreneurs (youth who either farm or trade in agricultural products, as their main occupation) and non-agripreneurs (youth who are engaged in other sectors of the Beninese economy).

### 3.2 Method of data analysis

To analyse youth participation in agripreneurship in Benin, the study employed descriptive and inferential statistics and an econometric approach. The descriptive statistics involved means and frequencies of key characteristics of youth entrepreneurs in Benin. Further, the study used t-tests and chi-square tests to test for statistical significance of the differences between agripreneurs and non-agripreneurs and traders and farmers as regards their socioeconomic characteristics and access to institutional support services. Also, the study used analysis of variance to test for statistical significance of the differences between farmers who produced food crops only, cash crops only and both food crops and cash crops. The tukey-hsd technique was used to test for the differences in the means. A youth entrepreneur may have different choices of livelihoods. This study analyses factors influencing the youth in making the choices between: (1) agripreneurship and non-agripreneurship, (2) farming and trading agricultural products, and (3) producing cash crops, food crops, and both. The first and the second circumstances are characterized by binary choices. Used binary logit model to analyse the data associated with these choices.

The binary logit model can be expressed in Eq. (1) as follows:

(1) $$y_{i}=\beta x_{i}^{\prime }+\varepsilon_{i}\ldots$$

where *y*_*i*_ is the dependent variable which takes a value of 1 if the entrepreneur is an agripreneur for the decision between agripreneurship or not and a farmer for the decision to be a farmer or a trader; *X =* covariate of regressors (age, gender, household size, number of children, education, formalized, gross margin, group membership, access to training, access to credit and location of youth); β = parameter estimates; ε = error term which is assumed to be iid (independently and identically distributed) with mean = 0 and variance = δ^2^.

From the generic equation (equation 2), a probit or a logit model can be estimated. However, according to Greene (2012), a probit model is used when the dependent variable is normally distributed, whiles a logit model is used when otherwise. However, the logit model is often used due to its mathematical convenience. The logit model used in the study is given by Eq. (2):

(2) $$P\left(Y=\frac{1}{X}\right)=\;\frac{\text{exp}\left(\beta x^{\prime }\right)}{1+exp\left(\beta x^{\prime }\right)}=\;\text{Λ}\left(\beta x^{\prime }\right)$$

where the notation Λ (.) indicates the logistic cumulative distribution function.

The third choice involves alternatives more than two. This study used the multinomial logit model to analyse the data. Following (Greene, 2012), for the *ith* youth faced with *j* choices, the utility of choice *j* is given by Eq. (3):

(3) $$U_{ij}=Z_{ij}^{\prime }+\varepsilon_{ij}$$

The choice of the type of crop to produce, Eq. (3) translates to Eq. (4):

(4) $$\begin{array}{ll} \;Prob\;\left(U_{ij}>\;U_{ik}\right) & \text{for ​all ​k}\neq j \end{array}$$

If the decision-maker chooses alternative *j* in particular, it is assumed that *U*_*ij*_ is the maximum utility decision-maker *i* derives from choosing alternative *j*. The probability that alternative *j* is chosen is given by Eq. (4). The model can be operationalized by a parameter choice of distribution for disturbances. Let *Y*_*i*_ denote a random variable that indicates the choices made. If and only if the *J* disturbances are independently and identically distributed, then Eq. (5) is derived:

(5) $$Prob\;\left(Y_{j}=j\right)=\frac{\text{exp}\left(Z_{ij}^{\prime }\theta \right)}{\sum_{j=1}^{J}\text{exp}\left(Z_{ij}^{\prime }\theta \right)}$$

Let *Z*_*ij*_ *= (X*_*ij*_*, w*_*i*_*),* and *θ* conformably into $\left[\beta^{\prime },\;\alpha^{\prime }\right].\;$ *X*_*ij*_ varies across the choices and possibly across the decision-makers as well. *X*_*ij*_ represents the characteristics of the choices or alternatives. *W*_*i*_ represents the characteristics of the decision-maker and it is the same for all choices. Incorporating these assumptions into the model, Eq. (6) becomes:

(6) $$Prob\;\left(Y_{i}=j\right)=\;\frac{\text{exp}\left(\beta X_{ij}^{\prime }\;+\alpha \;w_{i}^{\prime }\right)}{\sum_{j=1}^{J}\left(\beta X_{ij}^{\prime }\;+\;\alpha w_{i}^{\prime }\right)}$$

Eq. (6) is the multinomial logit model. A generic specification of the multinomial logit model is represented by Eq. (7):

(7) $$Prob\;\left(y_{j}=i\right)=\;P_{ij}\left(\beta_{0}+\beta_{1}\ldots \beta_{k}x_{k}\right)=P_{ji}=P_{ji}\left(\beta_{0}+\beta X\right)$$

Y_j_ is the probability of farmer j choosing alternative i (food crop only, cash crop only, or both food crop and cash crop)

*X*_*i =*_ covariate of regressors
β_i_ = the vector of coefficients associated with the crop choice

Table 1 presents the description of variables used in the econometric models.

**Table 1:** Description and measurement of explanatory variables.

Variable	Description	Measurement	Hypothesized signs		
			Entrepreneurship type	Agripreneurship type	Farmer type
Location	Whether the youth is located in a rural or urban area	Dummy (1 = urban; 0 = rural)	-	-	+/-
Gender	Gender of respondent	Dummy (1 = male; 0 = female)	+	+	+/-
Household size	Total members in the household including the youth	Continuous	+	+	+/-
Number of children	Number of children the youth has	continuous	+	+	+/-
Age	Age of youth in years	Continuous	+	+	+/-
Education	Whether the youth has received formal education	Dummy (1 = yes; 0 = no)	-	-	+/-
Formalized	Whether the current business of youth is registered	Dummy (1 = yes; 0 = no)	-	-	+/-
Group membership	Whether the youth belongs to a group	Dummy (1 = yes; 0 = no)	+/-	+/-	+/-
Training	Whether youth has received any training on the field of engagement	Dummy (1 = yes; 0 = no)	+/-	-	+/-
Gross margin	Monthly total revenue less total variable cost (CFA)	Continuous	-	-	+/-
Access to credit	Whether the youth has access to financial credit	Dummy (1 = yes; 0 = no)	-	-	+/-

## 4 Results

### 4.1 Descriptive statistics

The main agripreneurship activities in Benin include crop production (both food crops and cash crops) which was done on semi-subsistence or market-oriented levels, sale of agricultural inputs and sale of agricultural outputs. On the other hand, the non-agripreneurship activities are mainly in the areas of trading non-agricultural goods and artisanal works (such as dressmaking and masonry). The results are presented in Table 2.

**Table 2:** Characterization of entrepreneurs by entrepreneurship type (i.e., agripreneurs and non-agripreneurs).

Variable	Agripreneurs n = 338	Non-Agripreneurs n = 427		Pooled n = 765
**Continuous variables**			**t-value**	
Household size	8 (0.28)	7 (0.21)	-3.69∗∗∗	7 (0.17)
Number of children	1 (0.10)	1 (0.06)	-2.9186∗∗∗	1 (0.55)
Age	22.48 (0.24)	22.90 (0.20)	1.3508	22.72 (0.15)
Gross margin per month (CFA)	3503.15 (406.77)	4153.79 (446.81)	1.0510	3866.32 (307.44)
**Categorical variables**			**Chi**^**2**^**value**	
Gender (1 = Male)	45%	47%	0.2830	46%
Education (1 = Formal)	44%	72%	62.9521∗∗∗	60%
Formalized (1 = Yes)	6%	10%	4.3060∗∗	8%
Group membership (1 = Yes)	1%	9%	21.6465∗∗∗	5%
Training (1 = Yes)	4%	24%	63.4844∗∗∗	15%
Access to credit (1 = Yes)	11%	15%	1.7833	13%
Location (1 = urban)	53%	71%	27.6941∗∗∗	63%

**Notes:** Numbers in parentheses represent standard errors.

∗, ∗∗ and ∗∗∗ represent statistical significance at 10%, 5% and 1% level respectively.

1 West African CFA franc = USD$ 0.00168 at the time of the study.

The results show that the average age of youth entrepreneurs in Benin Republic is about 23 years. Agripreneurs were found to belong to larger households (8) compared with non-agriprenuers (7). Also, agripreneurs had more children than non-agripreneurs. More of the non-agripreneurs (72%) had access to formal education than the agripreneurs (44%). This is expected because most people who are engaged in agricultural activities do not have access to formal education. The results reveal that on average, a youth entrepreneur in Benin makes a monthly profit of CFA 3,866.32.

The results show that access to credit among entrepreneurs in Benin was only 13%. More of the non-agripreneurs had received institutional support services compared with the agripreneurs. The differences in access to these support services were statistically significant at the 1% level. The results show that the non-agripreneurs had an edge over the agripreneurs in terms of access to entrepreneurship training, membership to groups and the ability to register a business which was measured by having registered a business (formalized). The results also show that the proportion of non-agripreneurs who resided in urban areas was greater than that of the agripreneurs. This finding is intuitive given that agriculture and its related activities are predominantly undertaken in rural areas.

Generally, youth agripreneurs in Benin are either involved in crop production (farming) or trading as their main activities. The characteristics of these different agripreneurs are presented in Table 3. The average age of agripreneurs in Benin was 22 years. The results show that farmers had a larger household compared with traders. However, traders had more children than farmers. Although not strongly statistically significant, traders made more profits than farmers. This implies that trading as a main agripreneurship activity is more lucrative than farming in Benin Republic.

**Table 3:** Characterization of Agripreneurs by agripreneurship type (i.e., farmers and traders).

Variable	farmers n = 211	traders n = 127		Pooled n = 338
**Continuous variables**			**t-value**	
Household size	9 (0.39)	7 (0.31)	3.4977∗∗∗	8 (0.28)
Number of children	1 (0.10)	2 (0.20)	-1.9770∗∗	1 (0.95)
Age in years	22.3 (0.30)	22.7 (0.402143)	-0.9643	22.48 (0.24)
Gross margin per month (CFA)	2930 (508.50)	4455 (671.04)	-1.8223∗	3503.15 (406.77)
**Categorical variables**			**Chi**^**2**^**value**	
Gender (1 = Male)	60%	19%	54.6909∗∗∗	45%
Education (1 = Formal)	37%	55%	10.6121∗∗∗	44%
Formalized (1 = Yes)	6%	6%	0.0600	6%
Group membership (1 = Yes)	1%	1%	0.2728	1%
Training (1 = Yes)	2%	6%	4.4895∗∗	4%
Access to credit (1 = Yes)	4%	23%	27.3960∗∗∗	11%
Location (1 = urban)	42%	72%	28.54∗∗∗	53%

Notes: Numbers in parentheses represent standard errors.

∗, ∗∗ and ∗∗∗ represent statistical significance at 10%, 5% and 1% level respectively.

1 West African CFA franc = USD$ 0.00168 at the time of the study.

The results reveal that majority of farmers are males (60%) whereas the majority of traders are females (81%). Trading as an agripreneurship activity is female-dominated in Benin Republic. More of the traders than farmers had access to formal education. Only a few of the agripreneurs had registered their business (6%) and were members of groups (1%). The results show that the traders had an edge over the farmers in terms of access to entrepreneurship training and financial credit. Furthermore, the study shows that majority of the traders were in the urban areas whereas the majority of the farmers were in the rural areas. This shows that agricultural production is a predominant activity in rural areas whereas agricultural trading is a predominant activity in urban areas.

Table 4 presents the characteristics of youth farmers across the type of crop youth produced. The results show that household size varied across producers of food crops only and cash crops only. Youth farmers who produced cash crops only had a larger household size than youth who produced food crops only. This implies that youth who produce cash crop only has relatively more access to farm labour compared with youth who produced food crops only. Further, the results show that access to credit varied across the farmers, although very low among these farmers. More of the cash crop farmers (11%) had access to credit compared with the food crop farmers (3%). This shows that access to credit is very low among food crop farmers compared with cash crop farmers. Across all groups of farmers, it was revealed that the majority of youth who produced only cash crops (60%) were located in urban areas compared with those who produced food crops only and those who produced both food crops and cash crops. This implies that those who produced cash crops only had better access to urban markets, thereby increasing their propensity of selling to high-value markets and making more profits.

**Table 4:** Characterization of Farmer by type of crops produced.

Variable	Food crop (n = 137)	Cash crop (n = 47)	Both food and cash crop (n = 27)	F-value	Pooled (n = 211)
**Continuous variables**					
Household size	8 (4.64)^a,^∗	10 (7.31)	10 (7.03)	0.1028	9 (0.39)
Number of children	1 (1.38)	1 (1.49)	1 (1.21)	0.6926	1 (0.10)
Age in years	21.99 (4.23)	22.93 (4.37)	22.78 (4.62)	0.3609	22.3 (0.30)
Gross margin per month (CFA)	3009.1 (6580.8)	2274.8 (2989.27)	3669.7 (14033)	0.7222	2930 (508.50)
**Categorical variables**				**Chi**^**2**^**value**	
Gender (1 = Male)	60%	60%	63%	0.951	60%
Education (1 = Formal)	33%	43%	48%	0.215	37%
Formalized (1 = Yes)	8%	2%	4%	0.296	6%
Group membership (1 = Yes)	2%		4%	0.431	1%
Training (1 = Yes)	2%	2%	4%	0.730	2%
Access to credit (1 = Yes)	3%	11%		0.039∗∗	4%
Location (1 = urban)	37%	60%	33%	0.018∗∗	42%

**Notes:** Numbers in parentheses represent standard deviation.

∗ and ∗∗ represent statistical significance at 10% and 5% level respectively.

1 West African CFA franc = USD$ 0.00168 at the time of the study.

a = Cash crop only vs food crop only.

### 4.2 Challenges encountered by youth entrepreneurs

Table 5 presents the business challenges which the sample youth entrepreneurs reported. It shows that the challenges faced by youth entrepreneurs differ between non-agripreneurs and agripreneurs. The majority of youth entrepreneurs in Benin (31%) stated insufficient financial resources as their main challenge. The results suggest that scarcity of labour was a major challenge and even when labour was available, the youth were less qualified. Other challenges that the youth entrepreneurs encountered include market competition, insufficient business knowledge, scarcity of primary resources, and lack of access to technology. Financial constraint was more intense among agriprenuers than non-agripreneurs.

**Table 5:** Challenges encountered by youth entrepreneurs.

Challenges	Pooled n = 765	percent	Non-Agripreneurs n = 427	percent	Agripreneurs n = 338	percent
Insufficient financial resources	235	31	110	26	125	37
Poor personnel quality	15	2	5	1	10	3
Insufficient personal knowledge of the business	9	1.2	7	2	2	0.6
Scarcity of primary resources	11	1.4	5	1	6	2
Scarcity of labor	17	2.2	10	2	7	2
Technological access	1	0.1	0		1	0.3
Product development	9	1.2	1	0.2	8	2.4
Market competition	38	5	23	5	15	4
Other	430	56	266	62	164	48

Pearson Chi^2^ = 28.38 P = 0.0004.

### 4.3 Factors influencing entrepreneurship decisions among youth in Benin

Table 6 presents the results of the binary logit model. Columns (2) and (3) present results of the determinants of the choice between agriprenuership and entrepreneurship in other sectors of the economy (non-agrpreneurship) by youth entrepreneurs in Benin. The results show that the determinants of the choice between agripreneurship and non-agripreneurship include gender, age, education, household size, number of children, access to training, registered business, being a member of a group and the location of the youth (rural or urban area).

**Table 6:** Factors influencing entrepreneurship decisions among youth in Benin.

Variable	Marginal Effects	Coefficient	Robust Std. Err	P-values	Confidence intervals	
					Lower bound	Upper bound
Gender (male)	0.0749	0.3099	0.1761	0.0780	-0.0352	0.6550
Age	-0.0118	-0.0487	0.0215	0.0240	-0.0909	-0.0065
Formal education (yes)	-0.1947	-0.8054	0.1733	0.0000	-1.1451	-0.4658
Household size	0.01248	0.0516	0.0182	0.0050	0.0160	0.0873
Number of children	0.0263	0.1089	0.0636	0.0870	-0.0157	0.2335
Gross margin	-6.57e-07	-2.72e-06	9.47e-06	0.7740	-0.0000213	0.0000158
Training (yes)	-0.3742	-2.0248	0.3696	0.0000	-2.7492	-1.3003
Access to credit (yes)	-0.0398	-0.1666	0.2596	0.5210	-0.6754	0.3422
Registered (yes)	-0.0825	-0.3533	0.3164	0.2640	-0.9735	0.2668
Group membership (yes)	-0.2827	-1.4789	0.5571	0.0080	-2.5709	-0.3869
Location (urban)	-0.1073	-0.4411	0.1746	0.0120	-0.7833	-0.0989
Constant		1.3135	0.5319	0.0140	0.2710	2.3560
Observations	765					
LR $\chi^{2}$ (13)	97.7000					
Log likelihood	-446.0767					
Pseudo R^2^	0.1504					

**Notes:** Std. Err. Represents standard error.

Males are more likely to be agripreneurs than females. This is reflected in the positive relationship between gender and agripreneurship. Agriculture or agribusiness is predominantly undertaken by men and has been perceived as a male activity. This could be because women lag in access to information, advisory services and training, and productive resources such as land and agricultural technologies.

There exists a negative relationship between age and the decision to be an agripreneur. This shows that as youth grow older, the likelihood of choosing non-agricultural enterprises increases. This could be because, with time, youth accumulate the resources that can serve as capital for other businesses that are perceived to be more profitable and prestigious compared with agribusiness.

There exists a negative relationship between formal education and choosing agripreneurship which means that youth who have received formal education are less likely to venture into agribusiness and more likely to be entrepreneurs in other sectors of the economy. This implies that access to formal education deters youth from pursuing agribusiness and increases their tendency or propensity to pursue other options that appear to be more lucrative. This finding is consistent with the findings of Adeyanju et al. (2021), Ogunmodede et al. (2020), Ng'atigwa et al. (2020) and Ephrem et al. (2021) who found that more educated African youth are less likely to venture into agribusiness because youth perceive agribusiness as an occupation for less educated people.

The study also found a positive relationship between agripreneurship and household size; an increase in household size by one person increases the likelihood that youth would choose agribusiness over other businesses. This result is in line with the finding of Nnadi and Akwiwu (2008) who found that larger households necessitate agricultural production to meet food security needs. Further, the results show that the number of children a youth had was positively associated with the decision to participate in agribusiness.

There is a negative relationship between access to entrepreneurship training and agripreneurship. This could be attributed to inadequate agricultural or agribusiness training facilities in Benin. Adesina and Favour (2016) note that a major constraint to youth participation in agribusiness activities is limited agribusiness training facilities in rural areas in Sub-Saharan Africa. Even when entrepreneurship training facilities are available, youth are mostly in favour of non-agricultural entrepreneurship activities. Thus, youths who access these training are less likely to venture into agribusiness.

Collective action can influence the entrepreneurship decisions of youth in Benin. The study used group membership as a measure of collective action. The results show that there exists a negative correlation between youth participation in groups and the decision to engage in agripreneurship. This could be attributed to the limited rural youth engagement in collective action and the low attention given to the relevance of collective action and youth groups by development partners (Scoones et al., 2016). Further, studies have found that the limited engagement of rural youth in collective action and youth groups has led to the failure of interventions that seek to enhance youth participation in agribusiness activities (Amanor and Chichava, 2016; Lyocks et al., 2013). Even when youth participate in groups, differences in interests of stakeholders (such as development partners) and youth limit the performance of youth agribusiness ventures, thereby decreasing their propensity to increase participation in agribusiness activities.

The ability to register a business indicates the availability of educational, financial and technical capacities. The study found a negative association between having a registered business and the choice of agribusiness as an entrepreneurship activity.

Finally, as expected, youth who are located in the urban areas are more likely to be entrepreneurs in other sectors whereas those in rural areas are more likely to pursue agribusiness. This is because agriculture is a predominant activity in rural areas. This finding reinforces the need to invest in making agribusiness lucrative to attractive to the urban youth given that the results suggest that urban youth perceive agriculture or agribusiness as a rural activity.

### 4.4 Factors influencing the choice of different agribusiness activities

Table 7 presents the results of the determinants of the choice of different forms of agribusiness among youth in Benin. The results show that the factors that influence the choice of different agribusiness activities include gender, formal education, household size, access to formal financial services, access to credit and the location of the youth.

**Table 7:** Factors influencing the choice of different agribusiness activities.

Variable	Marginal effects	coefficient	Robust Std. Err	P-values	Confidence intervals	
					lower bound	upper bound
Gender (male)	0.4002	1.9828	0.3286	0.0000	1.3387	2.6269
Age	0.0061	0.0276	0.0379	0.4670	-0.0467	0.1019
Formal education (yes)	-0.2048	-0.9268	0.3011	0.0020	-1.5168	-0.3367
Household size	0.0185	0.0845	0.0305	0.0060	0.0248	0.1442
Number of children	-0.0051	-0.0233	0.0838	0.7810	-0.1875	0.1409
Gross margin	-1.44e-08	-6.55e-08	0.000019	0.9970	-0.00004	-0.00004
Training (yes)	-0.3019	-1.2530	1.0509	0.2330	-3.3129	0.8068
Access to credit (yes)	-0.3127	-1.3098	0.4522	0.0040	-2.1960	-0.4235
Registered (yes)	-0.0928	-0.4012	0.6381	0.5290	-1.6519	0.8494
Group membership (yes)	0.1590	0.8776	0.9634	0.3620	-1.0106	2.7658
Location (urban)	-0.2579	-1.2133	0.2946	0.0000	-1.7907	-0.6358
Constant		-0.1642	0.9019	0.8560	-1.9319	1.6035
Observations	338					
LR $\chi^{2}$ (13)	79.09					
Log likelihood	-162.3762					
Pseudo R^2^	0.2742					

The results show that males are more likely to be farmers whereas females are more likely to be traders. This could be because Beninese women as less likely than men to own land and even when youth have land, there was lower tenure security over such land (Goldstein et al., 2016). This reduces their propensity to engage in farming.

Formal education has a negative relationship with the choice of farming as an agribusiness activity by youth in Benin. This could be because of the wrong attitude of the youth towards farming. Many youths consider farming as an activity for uneducated people (Ng'atigwa et al., 2020). This reinforces the need to increase investments in agriculture to increase the financial returns of agriculture. This can enhance the image of agriculture, thereby attracting more youth into farming.

Household size is positively correlated with the choice of farming as an agribusiness activity. A large household leads to the intensification of the cultivation of land to meet the food security needs of the household (Muriithi and Matz, 2015), thereby encouraging youth participation in farming. Access to credit is negatively correlated with a youth's decision to be a farmer. This finding suggests that financial services drive youth to pursue trading activities which are more lucrative than farming activities. Access to financial services and credit increase the resource endowment of youth and hence, youth are capacitated to pursue trading activities. This finding is consistent with the findings of Beyene (2010) who found that access to credit increases participation in off-farm activities by rural households in Ethiopia.

Finally, the study found a negative relationship between the location of the youth agripreneur and the choice of agribusiness activity. The study found that youth who are located in the urban areas were less likely to be farmers and more likely to be traders. This finding was expected because trading activities are predominantly undertaken in the urban centres whereas farming activities mainly take place in the rural areas.

### 4.5 Factors that influence youth farmers’ crop choice decisions

Table 8 displays results of the multinomial logit model regarding farmers’ decision to grow cash crops, or food crops, or a combination of food and cash crops. The choice of food crops only has been taken as a base in the model. The results show that older youth are more likely to produce both food crops and cash crops and less likely to produce food crops only. Producing different types of crops is capital-intensive and requires experience. Since age is a proxy for experience and resource-endowment, older farmers can afford inputs required to produce different types of crops. Further, older youth have a higher propensity to be married which can induce them to produce both food and cash crops to meet household food security needs and to cover household expenses, respectively.

**Table 8:** Factors that influence youth farmers’ crop choice decisions.

Crop type	Coefficient	Marginal effects	p-value	Confidence interval	
				Lower bounds	Upper bounds
*Cash crop only*					
Gender	0.185 (0.39)	0.0376	0.639	-0.587	0.957
Age	0.072 (0.05)	0.0084	0.128	-0.02	0.164
Formal education	0.19 (0.42)	0.0317	0.65	-0.632	1.012
Formalized	-1.728 (1.38)	-0.2622	0.212	-4.442	0.985
Training	1.636 (1.206)	0.2363	0.175	-0.729	4.001
Gross margin	-2.47e-05 (3.29e-05)	-4.57E-06	0.377	-7.21e-05	2.73e-05
Household size	0.042 (0.03)	0.0044	0.215	-0.024	0.108
Number of children	-0.08 (1.15)	-0.0085	0.593	-0.376	0.215
Group membership	-14.077 (1.33)	-2.4071	0.000	-16.688	-11.466
Credit access	1.913 (0.92)	0.6855	0.038	0.103	3.723
Location: Urban	0.87 (0.41)	0.1333	0.035	0.061	1.678
Constant	-3.528 (1.14)		0.002	-5.756	-1.301
*Both food crops and cash crops*					
Gender	-0.286 (0.51)	-0.0271	0.576	-1.288	0.716
Age	0.091 (0.07)	0.0084	0.17	-0.039	0.222
Formal education	0.811 (0.51)	0.0670	0.113	-0.193	1.814
Formalized	-0.794 (0.90)	-0.0323	0.376	-2.55	0.962
Training	0.421 (1.13)	0.0069	0.71	-1.797	2.638
Gross margin	2.6e-05 (2.72-e05)	3.28e-06	0.334	2.72e-05	8.02e-05
Household size	0.076 (0.04)	0.0038	0.045	0.002	0.151
Number of children	-0.236 (0.21)	-0.0212	0.262	-0.648	0.176
Group membership	0.309 (1.15)	0.4298	0.788	-1.941	2.559
Credit access	-12.9 (0.94)	-1.4521	0.000	-14.748	-11.052
Location: Urban	-0.448 (0.47)	-0.0659	0.338	-1.364	0.468
Constant	-4.13		0.005	-7.034	-1.227
Observations	211				
McFadden's Pseudo R^2^	0.091				
McFadden's adjusted R^2^	0.103				
Prob > Chi^2^	0.0000				
Log pseudolikelihood	-168.37				

Notes: Numbers in parentheses represent robust standard errors. The base outcome is farmers who produced food crops only.

The youth having larger household sizes are more likely to produce both food crops and cash crops as compared to food crops only. Given that diversification is labour-intensive and that a large household implies the availability of labour, farmers who belong to larger households can meet the labour needs regarding producing both food crops and cash crops.

Access to credit is positively correlated with producing cash crops. This could be because farmers who have access to credit are more likely to afford the inputs that can enhance cash crop production. Given the lucrative nature of cash crops, access to credit can increase the resource-endowment of youth thereby creating a durable livelihood for this group of the population.

Youth in urban areas are more likely to specialize in cash crops production as compared to youth in rural areas. This could be because the urban-based youth have better access to markets arising from their proximity to the market.

## 5 Discussion

The study analysed the factors that influence youth entrepreneurs in Benin to participate in different entrepreneurship activities. The findings of the study revealed that generally, youth entrepreneurs in Benin would prefer to participate in entrepreneurship activities in other sectors of the economy but not agriculture. This finding corroborates the findings of Ephrem et al. (2021) who found that when youth are empowered in terms of education and access to institutional support services such as financial credit and training, their propensity of participating in agriculture significantly reduces. This finding also confirms a priori expectations given that the agricultural sector of developing countries is largely unattractive to young people (Akrong et al., 2020). However, in cases when young people participate in agriculture, agricultural trade was preferred to farming. This could be because, based on the descriptive statistics, agricultural trading is the most profitable agripreneurship activity among the sampled farmers. Further, with access to education and other institutional support services, youth in Benin would prefer to participate in trading activities in agriculture. However, youth entrepreneurs who have a large family size and more farming experience would prefer to be farmers. This implies that with knowledge about farming and with access to resources such as labour, young people have a higher propensity to venture into farming. This is intuitive given that farming in developing countries has been hypothesized to be labour-intensive which accounts for low youth participation (Adesina and Favour, 2016). Young farmers in Benin can choose to produce food crops only, cash crops only and both food crops and cash crops. It was hypothesized that cash crop production is the most lucrative farming activity in Benin. The findings of the study revealed that access to urban markets and financial credit could drive increased participation in cash crop production among youth in developing countries.

## 6 Conclusions and policy implications

With the increasing rate of unemployment, public-private partnerships supported self-employment and entrepreneurship initiatives have become common. Given the employment opportunities in the Beninese agricultural sector, youth in Benin are currently being encouraged to pursue entrepreneurship in agriculture. However, there is a lack of evidence on what drives the uptake of different entrepreneurship activities in agriculture. To this end, this study elucidated the factors that influence youth participation in different entrepreneurship activities in Benin, with a particular focus on agripreneurship. The results show that being a male, large family size and a large number of children encouraged entrepreneurs to venture into agribusiness whereas age, belonging to a group, ability to register a business, access to formal education and entrepreneurship training encouraged entrepreneurs to pursue non-agribusiness activities. Further, among participants of agribusiness, males and youth who belonged to larger households were more likely to be farmers whereas youth who were located in the urban areas, and had access to formal education, and financial credit was more likely to be traders. Finally, the study revealed that cash crop production by Beninese youth was highly motivated by access to financial credit and being located in urban areas. However, older Beninese youth and those who had a larger family size were more likely to produce both food crops and cash crops.

The study findings on the factors that influence youth to pursue agribusiness show that owing to the negative perception of youth about agriculture, support services such as training and access to social capital and collective action through group memberships can stimulate youth to shift to non-agricultural activities. Therefore, there is the need for capacity development programmes as well as agricultural training programmes. These programmes are necessary to enlighten Beninese youth on the potential of agribusiness to create a durable livelihood for them. Thus, with institutional support (such as belonging to a group, business registration or formalization, access to formal education, entrepreneurship training and credit facilities) rural youth would be encouraged to pursue a career in agribusiness. Further, the study recommends that youth Beninese should be provided with modern agricultural technologies as well as productivity-enhancing technologies. This will ensure that youth farmers maximize gains from agriculture, thereby making agriculture lucrative and attractive.

The findings suggest that pro-agribusiness programmes should target trading activities since agricultural trading was found to be the most profitable entrepreneurship activity to the youth in Benin. To ensure the sustainability of agricultural trading by youth, the government of Benin and development partners alike should promote formal education and capacity development programmes among youth in Benin. This will equip Beninese youth with the knowledge and skills required to ensure business success. Moreover, financial credit and formal financial services should be made available and accessible to these youths. This will increase their participation in trading activities as well as boost their profits. This will capacitate youth to meet business requirements as well as enable them to expand their businesses.

Given that availability of resources, wealth and experience, and labour, measured with age and household size, respectively, and access to financial credit encourage cash crop production among youth in Benin, it will be useful if the government of Benin and development partners alike can promote rural youth participation in such high-value crops by ensuring that financial resources are made available and accessible to youth. This will enable the youth to afford hired labor and improved inputs and technologies that can encourage and enhance cash crop production.

## Declarations

### Author contribution statement

Rexford Akrong: Conceived and designed the experiments; Analyzed and interpreted the data; Contributed reagents, materials, analysis tools or data; Wrote the paper.

Bekele Kotu Hundie: Conceived and designed the experiments; Analyzed and interpreted the data; Contributed reagents, materials, analysis tools or data.

### Funding statement

This work was supported by the 10.13039/100008687International Fund for Agricultural Development.

### Data availability statement

Data associated with this study has been deposited at ILO Statistics (https://www.ilo.org/employment/areas/WCMS_234860/lang--en/index.htm).

### Declaration of interests statement

The authors declare no conflict of interest.

### Additional information

No additional information is available for this paper.
